# Exploring the potential anti-senescence effects of soybean-derived peptide Soymetide in mice hippocampal neurons via the Wnt/β-catenin pathway

**DOI:** 10.3389/fphar.2025.1510337

**Published:** 2025-02-25

**Authors:** Asmita Garg, Jyotshana Saroj, Saurabh Tiwari, Uttara Das, Neetu Shukla, Jimut Kanti Ghosh, Sanghamitra Bandyopadhyay

**Affiliations:** ^1^ Systems Toxicology Group, Food, Drug and Chemical, Environment and Systems Toxicology Division, CSIR-Indian Institute of Toxicology Research (CSIR-IITR), Lucknow, Uttar Pradesh, India; ^2^ Academy of Scientific and Innovative Research (AcSIR), Ghaziabad, India; ^3^ Biochemistry and Structural Biology Division, CSIR-Central Drug Research Institute, Lucknow, India

**Keywords:** Soymetide, senescence, doxorubicin, Wnt/β-catenin pathway, hippocampus, neuroprotection

## Abstract

Soybean-based foods enhance cognitive functions by influencing hippocampal mechanisms. These salutary effects have so far been attributed to isoflavones present in soybeans. Considering cellular senescence contributes to cognitive decline and that no specific soy-derived peptides are known for their potential to mitigate senescence, we examined the efficacy of a thirteen amino acid soy-derived peptide, Soymetide, on a doxorubicin-induced senescence mice model. Soymetide pretreatment lowered the senescence markers p53, p21 and p16, pro-inflammatory cytokines, and Senescence β-Galactosidase staining while enhancing the mature neuronal marker NeuN in the hippocampus. This anti-senescent effect was comparable with that of a well-known senolytic combination (dasatinib and quercetin). Research indicates that Wnt signaling influences cellular senescence, and our findings here demonstrate that doxorubicin decreased hippocampal Wnt3a, p-LRP6, Frizzled, Dishevelled, Axin1, and β-catenin levels and increased GSK-3β, while Soymetide mitigated these effects. Additionally, upon inhibition of the Wnt/β-catenin pathway, Soymetide’s ability to reduce senescence markers and restore NeuN expression was reduced. We validated the anti-senescence impact on hippocampal neurons by co-immunostaining Wnt/β-catenin and senescence indicators alongside NeuN in mice and assessed it in primary hippocampal neurons. Further examining the neuronal survival and functions revealed that Soymetide blocked the doxorubicin-induced loss in Nissl-stained surviving neurons and learning-memory performances, measured by Y-Maze and Passive Avoidance tests, which Wnt/β-catenin inhibitors could counteract. In conclusion, our study identifies a novel Wnt/β-catenin-linked mechanism of doxorubicin-induced senescence in the hippocampal neurons and demonstrates Soymetide’s effectiveness in reversing this process. Hence, this suggests Soymetide’s potential therapeutic application in addressing cognitive decline associated with cellular aging.

## Introduction

Cellular senescence, characterized by irreversible cell cycle arrest, is increasingly recognized for its role in hippocampal decline (Lin et al., 2021). Senescence affects hippocampal neurons by diminishing their functionality and plasticity, leading to a decline in cognitive abilities such as learning and memory (Miyamoto et al., 1986). The senescent neurons express markers like p53, p21, p16, and β-Galactosidase and morphological alterations (Mijit et al., 2020; Wang et al., 2024). This may promote the generation of senescence-associated secretory phenotype (SASP) cytokines, exacerbating neuroinflammation (Saito et al., 2021). Hence, while cellular senescence is a physiological process, its aberrant accumulation adversely affects neuronal health, causing age-associated hippocampal dysfunction (Piechota et al., 2016; Wissler Gerdes et al., 2020).

The Wnt signaling pathway, particularly involving Wnt3a, plays a crucial role in hippocampal neuronal processes and functions (Fortress and Frick, 2016; Arrazola et al., 2017). An activated Wnt pathway has been linked to enhanced neurogenesis and neuronal survival within the hippocampus (Inestrosa et al., 2004; Avila et al., 2010; Wei et al., 2012; Zhang et al., 2022a; Tao et al., 2023). Additionally, reduced Wnt3a expression or aberrant downstream signaling events may contribute to cognitive decline (Oliva et al., 2013; Palomer et al., 2019). Wnt3a interacts with cell-surface receptor Frizzled (Fz) and Low-density lipoprotein receptor-related protein 6 (LRP6), triggering downstream events involving Dishevelled (Dvl) and Axin1 (Huang and Klein, 2004; Garg and Bandyopadhyay, 2024). Notably, Glycogen Synthase Kinase-3β (GSK-3β) serves as a key player in the Wnt pathway, where an inhibited GSK-3β may lead to cytoplasmic accumulation of β-catenin, affecting hippocampal functions and neuronal health (Beurel et al., 2015; Li et al., 2024).

Emerging evidence suggests a link between the Wnt/β-catenin signaling pathway and cell senescence or aging in the brain (Jia et al., 2019; Chen et al., 2020). A dysregulated Wnt signaling associated with senescence involving morphological changes in brain cells, cell cycle alterations, β-Galactosidase staining, and decline in cognitive functions (Li et al., 2018; Swarbrick et al., 2019; Xiang et al., 2019; Zhang et al., 2019; Prud’homme et al., 2022). Cellular senescence demonstrated a connection with Wnt signaling in the hippocampus as well, involving the participation of altered Dickkopf-1 (a secreted inhibitor of β-catenin-dependent Wnt signaling), GSK-3β activation, β-catenin, along with neuronal loss and dysregulated expression of apoptosis-related proteins (Bayod et al., 2015). Hence, targeting Wnt pathway components and reducing senescence could hold promise as a therapeutic strategy for mitigating neurodegeneration and cognitive decline.

The beneficial effects of soy-based foods in mitigating cognitive decline are primarily attributed to phytoestrogen isoflavones (Kim, 2021). Although largely uncharacterized, research has shown promising outcomes in animal models of cognitive decline and behavioral deficits with soy protein isolates/peptide mixture (Dong et al., 2023; Zheng et al., 2023; Tamura et al., 2024). These protein isolates/peptide mixtures/soy food demonstrated protection against neuronal damage, promoted neurogenesis, regulated neurotransmitter synthesis and metabolism, increased neurotrophin signaling, induced anti-inflammatory and antioxidant effects in the brain, modulated neuronal apoptosis, and mitigated cognitive decline (Jeong et al., 2021; Dong et al., 2023; Zheng et al., 2023; Tamura et al., 2024). Human studies also indicate a lower risk of dementia and significant improvements in cognition and neurotrophin levels associated with consuming soy products (Hwang et al., 2019; Deng et al., 2023).

Building on this evidence, our study aims to investigate the therapeutic potential of a short-length soy-derived bioactive peptide, Soymetide (Schuler et al., 1982; Tsuruki et al., 2003) in preventing age-related senescence. Utilizing a doxorubicin-induced senescent mouse model, we explored the effects of Soymetide on the senescence markers in the hippocampus. We investigated the participation of the Wnt/β-catenin pathway in the process. Our study aimed to examine the anti-senescence and cognitive improvement capabilities, as well as the underlying mechanisms of Soymetide for potential therapeutic applications in senescence-related neurological disorders of which memory impairment is the dominant type. Through the investigation of these mechanisms, we seek to evaluate Soymetide’s efficacy in improving cognitive function and its therapeutic role in addressing age-related neurological conditions, especially those associated with memory deficits.

## Materials and methods

### Reagents, chemicals and antibodies

Doxorubicin hydrochloride (cat no. PHR1789), dasatinib (cat no. SML2589), quercetin (cat no. Q4951), β-Catenin/Tcf Inhibitor III, iCRT3 (cat no. 219332) and cresyl violet acetate (cat no. C5042) were bought from Sigma-Aldrich (St. Louis, Missouri). Human Dkk-1 Recombinant Protein (cat no. PHC9214) and Mouse ProcartaPlex Mix&Match 9-plex kit (cat no. PPX-09-MX2XAC3) were purchased from Thermo Fisher Scientific (Waltham, Massachusetts). Senescence β-Galactosidase Staining Kit (cat no. 9860) was purchased from Cell Signaling Technology (Danvers, Massachusetts). Polyvinylidene difluoride membrane (cat no. IPVH00010) and Immobilon Western Chemiluminescent HRP Substrate (cat no. WBKLS0500) were bought from MilliporeSigma (Burlington, Massachusetts). The prestained protein ladders (cat no. PG-PMT2922 and cat no. 786419) were procured from Genetix Biotech Asia Pvt Ltd. (New Delhi, India) and G-Biosciences (St. Louis, Missouri), respectively. Vectashield mounting medium with DAPI was purchased from Vector Laboratories (Newark, California). Mouse monoclonal p53 (cat no. 2524), rabbit polyclonal p21 (cat no. 64016), phospho-LRP6 (cat no. 2568), β-catenin (cat no. 9562), rabbit monoclonal NeuN (cat no. 24307), LRP6 (cat no. 3395), Dvl2 (cat no. 3224), Axin1 (cat no. 2087), GSK-3β (cat no. 12456) and TCF3/TCF7L1 (cat no. 2883) antibodies were purchased from Cell Signaling Technology. Rabbit polyclonal p16INK4a (cat no. PA5-20379) antibody was purchased from Thermo Fisher Scientific. Rabbit polyclonal Wnt3a (cat no. 09162), HRP-conjugated secondary anti-rabbit IgG (cat no. A0545), and anti-mouse IgG (cat no. A9044) antibodies were purchased from Sigma-Aldrich. Rabbit polyclonal Frizzled-7 (cat no. ab64636) antibody was purchased from Abcam (Cambridge, Massachusetts). Horseradish peroxidase (HRP)-conjugated mouse monoclonal β-actin (cat no. sc-47778) antibody was procured from Santa Cruz Biotechnology (Dallas, Texas). Mouse monoclonal NeuN (cat no. 66836-1-lg) and rabbit polyclonal NeuN (cat no. 26975-1-AP) antibodies were procured from Proteintech (Rosemond, Illinois). Alexa Fluor^®^546 goat anti-rabbit IgG (cat no. A11010), Alexa Fluor^®^488 goat anti-mouse IgG (cat no. A11001), Alexa Fluor^®^546 goat anti-mouse IgG (cat no. A11030) and Alexa Fluor^®^488 goat anti-rabbit IgG (cat no. A11008) were procured from Invitrogen (Carlsbad, California). *In Situ* Cell Death Detection Kit, Fluorescein (cat no. 11684795910) was purchased from Roche (Mannheim, Germany).

### Peptide synthesis

Soymetide (Soym; MITLAIPVNKPGR) (Schuler et al., 1982; Tsuruki et al., 2003) was synthesized (98.27% purity (HPLC), −20°C storage in the dark) by GL Biochem Shanghai Ltd. (Shanghai, China). We designed the Scrambled peptide (Scr; MVNLGIPITKPAR), which was subsequently synthesized by GL Biochem Shanghai Ltd.

### Animal treatments

C57BL/6 male mice were kept under a 12-h light/dark cycle condition with *ad libitum* availability of chow diet and R. O water. The treatment and assessment timeline of mice is as shown in Supplementary Material 1. To generate a senescence mice model, mice (≅ 22-25 gm) were intraperitoneally injected with doxorubicin hydrochloride (DOX; 1, 5 and 10 mg/kg in PBS), once a week for 4 weeks, closely following the reported protocol (Sun et al., 2022). To examine whether Soymetide had anti-senescence properties, it was once injected bilaterally (2 μL/min) into the hippocampus of mice through stereotaxic surgery (coordinates: AP −1.7, ML +1.6, DV −1.9 to bregma) (Sierra et al., 2015; Garg and Bandyopadhyay, 2024) at doses of 10, 50, and 100 μg/kg in PBS (4 µL), 7 days before the doxorubicin treatment. To specifically identify the effect of Soymetide, scrambled peptide (50 μg/kg in PBS) was given to the mice in the same method and schedule. A senolytic combination of dasatinib (D) (5 mg/kg) plus quercetin (Q) (50 mg/kg) (D + Q) (Wang et al., 2023) was orally administered to the doxorubicin-treated mice once a week for 4 weeks as a positive control. To assess the role of Wnt in the doxorubicin and Soymetide-induced effects, recombinant Dickkopf-1 (rDkk1) protein [200 ng in 2 µL sterile saline (Garg and Bandyopadhyay, 2024)] was injected into the hippocampus of Soymetide- and doxorubicin-treated mice through stereotaxic surgery, 7 days before the assessment. To assess the role of β-catenin, inhibitor of β-catenin responsive transcription-3 (iCRT3) protein [5 mg/kg in sterile saline with 5% DMSO (Sharma et al., 2017)] was injected intraperitoneally into the Soymetide- and doxorubicin-treated mice. Vehicle-only-treated sets were included for every treatment. Animal treatment and behavioral analyses were randomized to ensure an unbiased approach.

### Western blotting

The hippocampal tissue was isolated from the mice brain, and the proteins (30–50 μg) were separated using SDS-PAGE (8%–15%) and transferred onto a PVDF membrane, as we reported (Garg and Bandyopadhyay, 2024). The blots were probed with p53, p21, p16, NeuN, Wnt3a, p-LRP6, LRP6, Fz, Dishevelled, Axin1, GSK-3β, β-catenin and TCF3 antibodies for overnight (1:1000 dilution; 4°C), and HRP-conjugated β-actin antibody (loading control) for 2 h (1:5000; room temperature). Except for the β-actin-probing, the blots were incubated with HRP-conjugated secondary antibody for 2 h (1:5000 dilution; room temperature) and developed using Immobilon Western Chemiluminescent HRP Substrate in Amersham Imager 600 (GE Healthcare Life Sciences, Pittsburgh, Pennsylvania). Relative protein levels were determined by densitometric quantification by applying the Quantity One software (Bio-Rad Laboratories, Hercules, California).

### Cytokine multiplexing

Blood samples of mice were collected and serum was isolated (Pandey et al., 2022). The quantitative determination of cytokine levels in serum was performed using the Mouse ProcartaPlex Mix&Match 9-plex kit as per its manufacturer’s protocol. The cytokine levels were measured using Luminex MAGPIX^®^ instrument (Bio-Rad Laboratories) and expressed as the mean Magnetic Fluorescence Intensity (MFI).

### Senescence β-galactosidase staining

The whole brain was isolated from mice after transcardial perfusion with PBS, cryoprotected (on dry ice) in OCT and stored at −80°C (Das et al., 2016), and subsequently cut into 30-μm thick coronal sections using a cryomicrotome (Microm HM 520, Labcon, Germany). Following the manufacturer’s protocol of the Senescence β-Galactosidase staining kit, the sections were fixed (using the fixative solution) for 15 min at room temperature, stained with β-Galactosidase staining solution (pH 6.0) that contained 0.2% X-Gal, and incubated for 18 h at 37°C in a dry incubator (Sarkar et al., 2011). The sections were then mounted with 70% glycerol solution, and images of the hippocampus were captured under a 20× objective [Leica DMi1 microscope (Leica Microsystems, Wetzlar, Germany)]. The results were quantified using ImageJ software (Wayne Rasband, NIH).

### Immunofluorescence

The whole brain was isolated from mice after perfusion with PFA (4%), cryoprotected, and 10-µm thick sections were prepared using a cryomicrotome, as previously described by us (Gupta et al., 2022). Sections were then probed with the Wnt3a, β-catenin, p53, p16, and NeuN primary antibodies overnight (1:250 dilution; 4°C), incubated with Alexa Fluor secondary antibodies for 2 h (1:500 dilution; room temperature), and mounted using VECTASHEILD anti-fade mounting media containing DAPI. Fluorescence photomicrography of the hippocampus was conducted in these sections under 20× objective (NIS-Elements software, Nikon Instech Co. Ltd., Kawasaki, Kanagawa, Japan), and the images were processed using the ImageJ software.

### Nissl staining

Cryosections of 30-µm thickness were made and processed for Cresyl violet acetate staining following our previously described protocol (Mishra et al., 2021). Images of the hippocampus were captured in these sections with 20× and 40× objectives [Leica DMi1 microscope (Leica Microsystems)] and imported into the ImageJ software. The surviving neurons (%) were then manually counted from five different fields using the Cell Counter plugin.

### Terminal deoxynucleotidyl transferase dUTP nick-end labeling (TUNEL) assay

Hippocampal neuronal apoptosis was assessed as reported earlier (Pandey et al., 2017). Briefly, 10-µm brain sections were treated with TdT and fluorescein-labeled dUTP, immunostained with anti-rabbit NeuN, and then mounted. The images of the hippocampus were captured (20×) and TUNEL-positive neurons were counted in five random areas using ImageJ, expressed as TUNEL-positive cells per 100 DAPI-stained nuclei.

### Learning and memory assays

Mice were subjected to Y-maze and Passive Avoidance tests to assess learning and memory functions, as we previously reported (Pandey et al., 2020). For the Y-Maze test, the learning trials (training) were conducted on mice in a Y-Maze apparatus, where running to the unsafe arms (dark arms with foot shock) from the safe arm (bright and shock-free) was considered an error. The % number of errors (E) was calculated for learning trials. Further, the % Saving Memory was assessed in the Y-Maze apparatus at 24 h, 48 h, and 7th day post-learning, and calculated as (Etraining−Etest)×100/Etraining. During the Passive Avoidance test, mice were subjected to acquisition trials in a gated two-compartment apparatus. One compartment was bright and free of shocks, while the other was dark and delivered an electrical shock. After 24 h (R1), 48 h (R2), and 72 h (R3) of acquisition trials, three shock-free retention trials of 300 s each were conducted. The time taken by the mice to move from the bright to the dark compartment was considered as transfer latency time (TLT) for learning-memory performance.

### Primary neuronal culture and treatments

Embryos were collected at embryonic day 16 (E−16), and the hippocampus was dissected in Hank’s Balanced Salt Solution (HBSS), followed by mechanical digestion in serum-free neurobasal media, as previously reported (Pandey et al., 2020). The suspension was digested using 0.05% trypsin-EDTA and incubated for 15 min at 37°C in a CO_2_ incubator, and treated with trypsin inhibitor to neutralize the effect of trypsin. The remaining suspension was centrifuged at 1500 rpm for 10 min and the pellet was suspended in the complete neurobasal medium containing N2 supplement (1%), B-27 supplement (2%), 2 mM L-glutamine, penicillin (100 units/mL), streptomycin (100 μg/mL) and amphotericin (0.25 μg/mL). Cells were plated on poly-L-lysine-coated 25 cm^2^ flask or 4-well culture plates and grown in a humidified incubator at 37°C with 5% CO_2_. Doxorubicin at 50 nM (Marques et al., 2020) was then added to the neurons for 18 h to induce senescence. For standardizing the treatment dose of Soymetide, neurons were pre-treated for 2 h with Soymetide at doses of 1, 10, and 100 nM followed by co-treatment with doxorubicin and assessed for the lowest dose affecting p53 levels (Supplementary Material 2). To analyze the role of Wnt and β-catenin, rDkk1 2.38 nM (Wei et al., 2019) and iCRT3 20 μM (Lee et al., 2013) were given to the cells, respectively, 2 h before the assessment time.

### Protein extraction and western blotting (*in vitro*)

Primary neurons were first lysed with cell lysis reagent and Western blotting was carried out as above using the Wnt3a, β-catenin, p53, p16, NeuN and β-actin antibodies.

### Senescence β-galactosidase staining (*in vitro*)

Primary hippocampal neurons were plated onto poly-L-lysine-coated chamber slides. Following the treatment and washing with PBS, fixation, and staining of the cells were performed as per the manufacturer’s protocol of the Senescence β-Galactosidase staining kit. Briefly, the cells were fixed in the fixative solution for 10–15 min at room temperature, washed with PBS, and incubated in a no CO_2_ incubator with the staining solution overnight at 37°C. 70% glycerol solution was used to mount the slides. Images of the cells were captured with a 40× objective [Leica DMi1 microscope (Leica Microsystems)].

### Statistics

**FIGURE 1:**
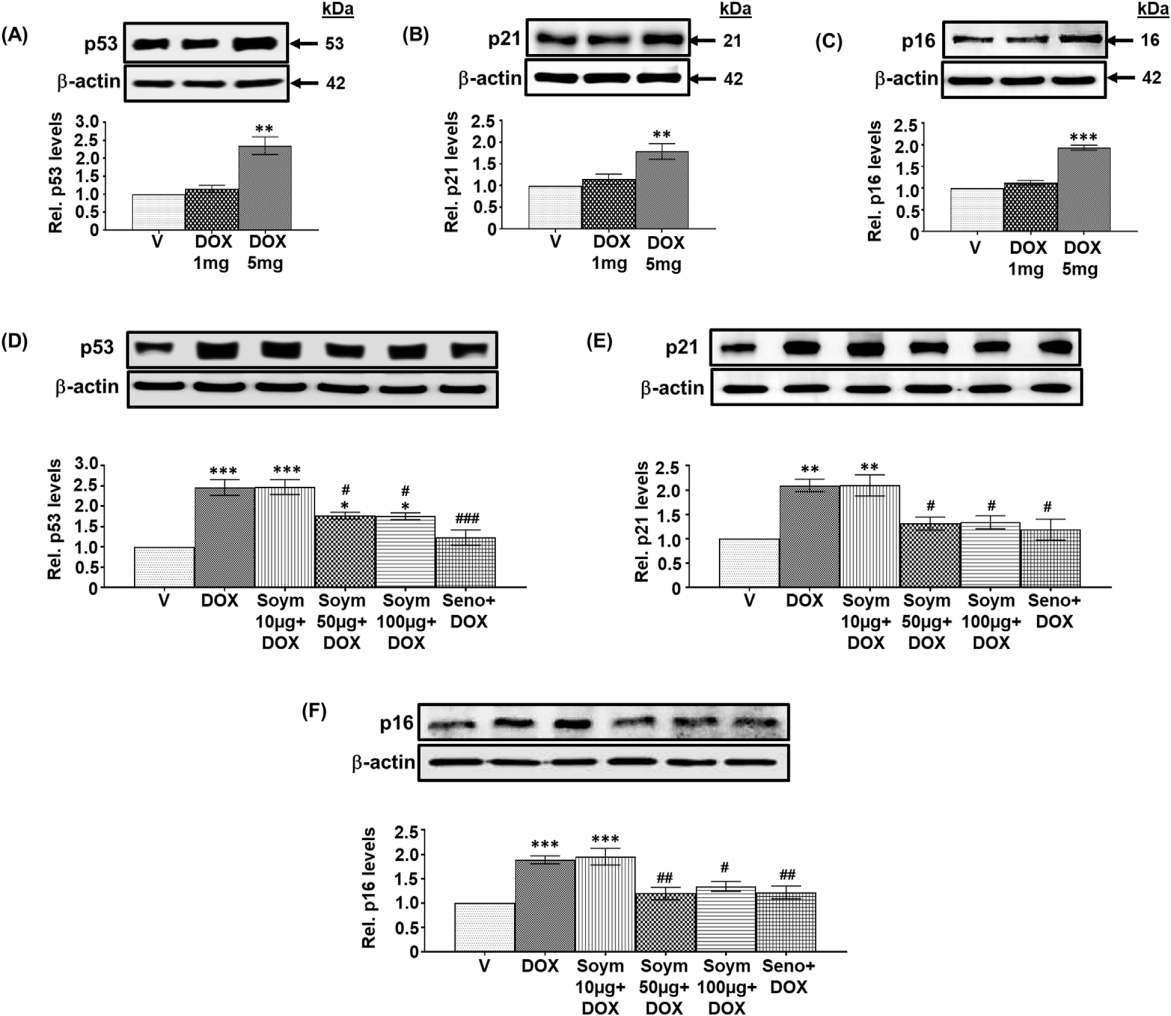
*Soymetide prevents doxorubicin-induced senescence in the hippocampus of mice*. Hippocampal tissues were isolated from vehicle (V), doxorubicin (DOX), Soymetide (Soym)+DOX and senolytic combination (Seno)+DOX-treated mice. **(A–F)** Representative Western blots and corresponding densitometry (relative to (Rel.) vehicle) of p53 **(A, D)**, p21 **(B, E)**, and p16 **(C, F)** normalized with β-actin in the hippocampal tissues. Data represent means ± Standard Error (SE) of three mice/group. ***p < 0.001, **p < 0.01 and *p < 0.05 compared to V. ^###^p < 0.001, ^##^p < 0.01 and ^#^p < 0.05 compared to DOX.

**FIGURE 2:**
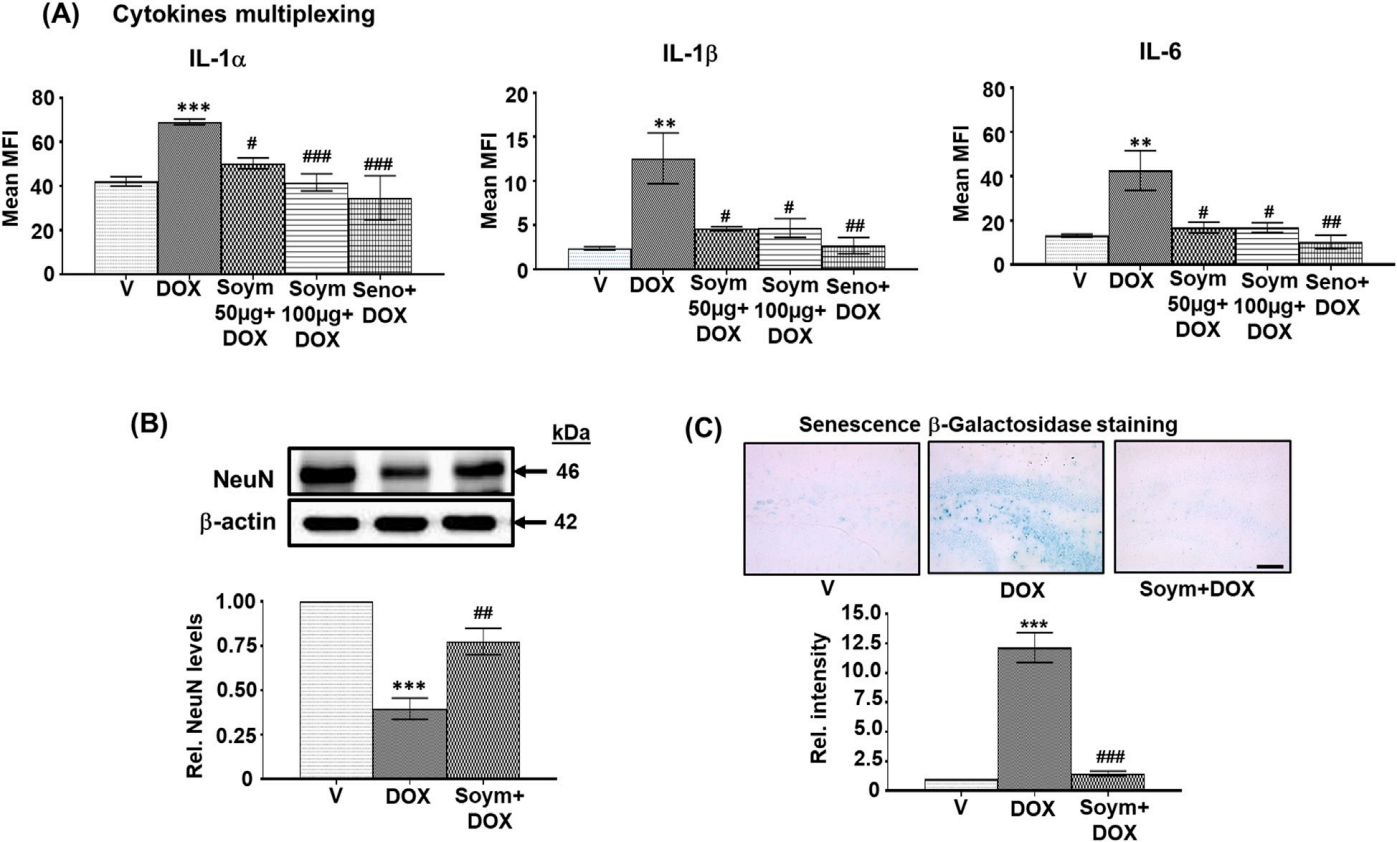
*Soymetide prevents doxorubicin-induced rise in serum cytokine levels and hippocampal β-Galactosidase staining and the loss of hippocampal NeuN levels in mice*. Serum was isolated from the blood of vehicle (V), doxorubicin (DOX), Soymetide (Soym)+DOX, and senolytic combination (Seno)+DOX-treated mice. **(A)** Representative densitometry of serum IL-1α, IL-1β and IL-6 levels. Hippocampal tissues and whole brain were isolated from vehicle (V), doxorubicin (DOX), and Soymetide (Soym)+DOX-treated mice. **(B)** Representative Western blots and corresponding densitometry (relative to vehicle) of NeuN normalized with β-actin in the hippocampal tissues. **(C)** Representative photomicrographs and corresponding bar graph (relative to vehicle) of Senescence β-Galactosidase-stained hippocampus. Scale bar: 100 µm. Data represent means ± SE of three mice/group. ***p < 0.001 and **p < 0.01 compared to V. ^###^p < 0.001, ^##^p < 0.01 and ^#^p < 0.05 compared to DOX.

**FIGURE 3:**
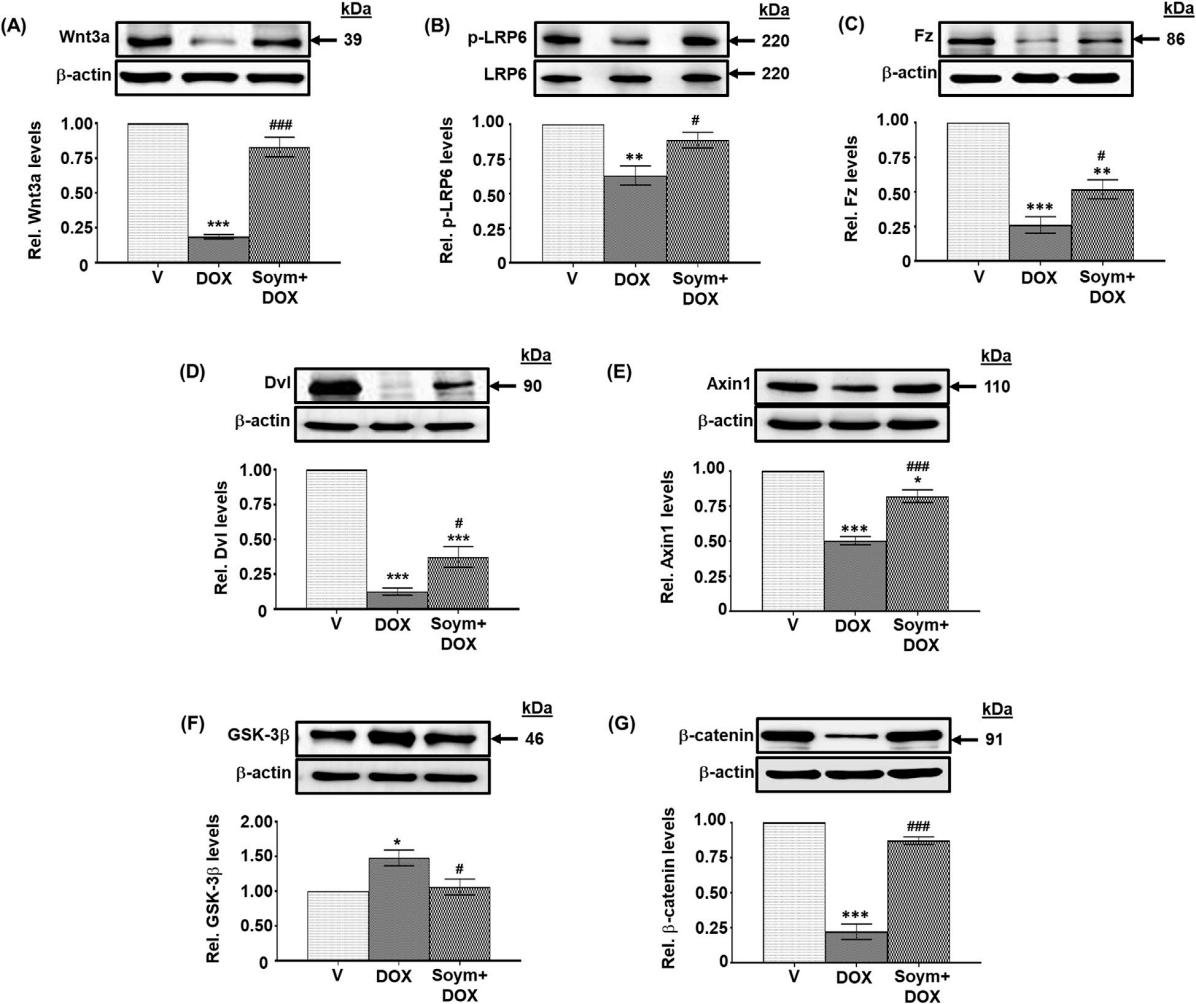
*Soymetide inhibits doxorubicin-induced attenuation of hippocampal Wnt3a/β-catenin pathway in mice*. Hippocampal tissues were isolated from vehicle (V), doxorubicin (DOX) and Soymetide (Soym)+DOX-treated mice. **(A–G)** Representative Western blots and corresponding densitometry (relative to vehicle) of Wnt3a **(A)**, p-LRP6 **(B)**, Frizzled (Fz) **(C)**, Dishevelled (Dvl) **(D)**, Axin1 **(E)**, GSK-3β **(F)** and β-catenin **(G)** normalized with β-actin **(A, C–G)** or LRP6 **(B)** in the hippocampal tissues. Data represent means ± SE of three mice/group. ***p < 0.001, **p < 0.01 and *p < 0.05 compared to V. ^###^p < 0.001 and ^#^p < 0.05 compared to DOX.

**FIGURE 4:**
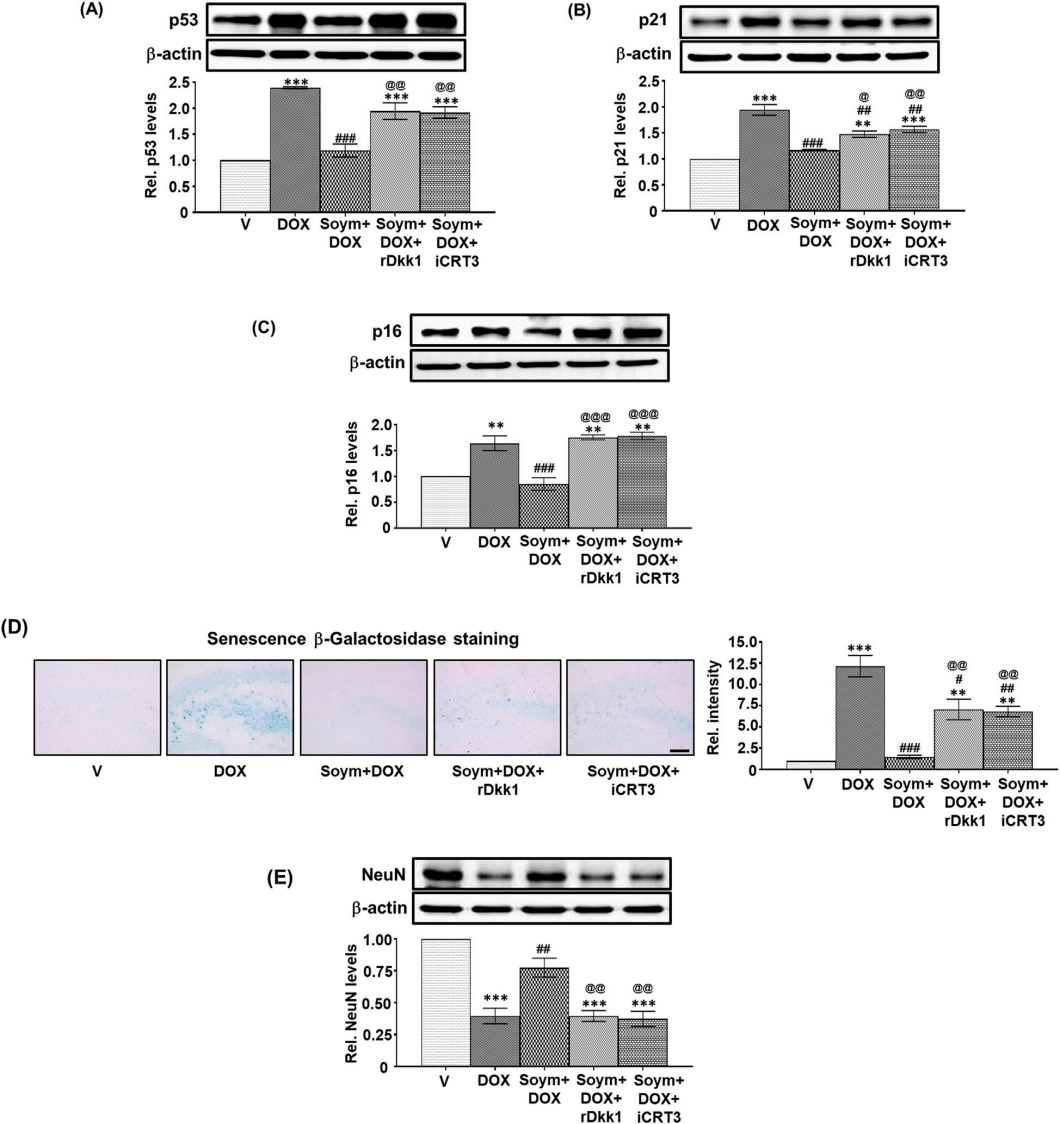
*The Soymetide-induced reduction in senescence markers and increase in NeuN levels are Wnt3a/β-catenin-dependent in the hippocampus of doxorubicin-treated mice*. Hippocampal tissues and the whole brain were isolated from vehicle (V), doxorubicin (DOX), Soymetide (Soym)+DOX, Soym + DOX + rDkk1, and Soym + DOX + iCRT3-treated mice. **(A–C, E)** Representative Western blots and corresponding densitometry (relative to vehicle) of p53 **(A)**, p21 **(B)**, p16 **(C)** and NeuN **(E)** normalized with β-actin in the hippocampal tissues. **(D)** Representative photomicrographs and corresponding bar graph (relative to vehicle) of Senescence β-Galactosidase-stained hippocampus in brain sections. Scale bar: 100 µm. Data represent means ± SE of three mice/group. The quantification of the first three groups in **(D)** and **(E)** are based on the same data sets as in Figures 2B, C. ***p < 0.001 and **p < 0.01 compared to V. ^###^p < 0.001, ^##^p < 0.01 and ^#^p < 0.05 compared to DOX. ^@@@^p < 0.001, ^@@^p < 0.01 and ^@^p < 0.05 compared to Soym + DOX.

**FIGURE 5:**
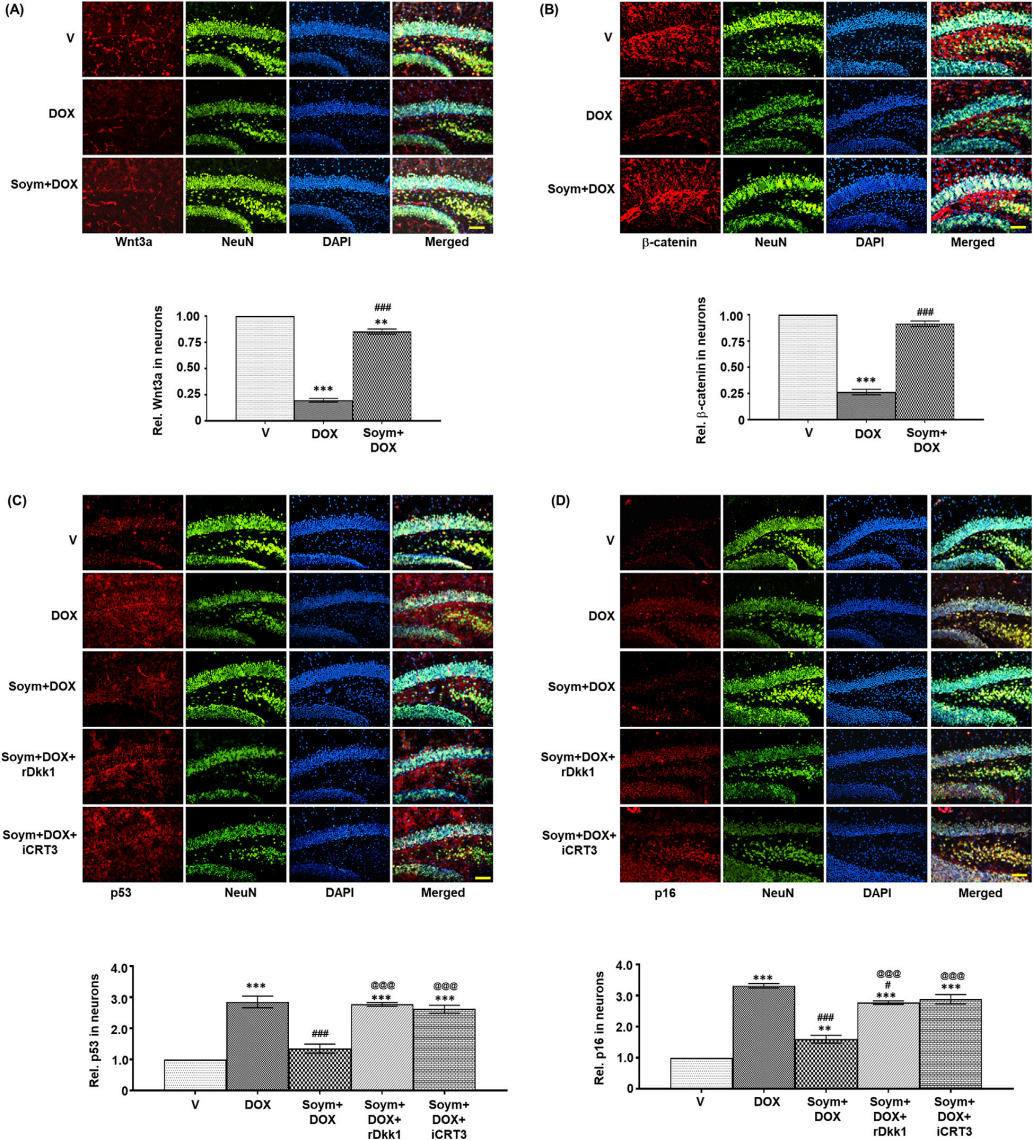
*Soymetide increases Wnt3a and β-catenin and decreases p53 and p16 levels in the hippocampal neurons of doxorubicin-treated mice*. Fluorescence immunohistochemistry was performed on brain sections of vehicle (V), doxorubicin (DOX), Soymetide (Soym)+DOX, Soym + DOX + rDkk1 and Soym + DOX + iCRT3-treated mice. **(A–D)** Representative fluorescence photomicrographs of Wnt3a **(A)**, β-catenin **(B)**, p53 **(C)** and p16 **(D)** co-immunolabeled with NeuN and counter-stained with DAPI in the hippocampus of brain sections. Bar diagrams represent corresponding quantification relative to vehicle. Scale bar: 100 µm. Data represent means ± SE of three mice/group. ***p < 0.001 and **p < 0.01 compared to V. ^###^p < 0.001 and ^#^p < 0.05 compared to DOX. ^@@@^p < 0.001 compared to Soym + DOX.

**FIGURE 6:**
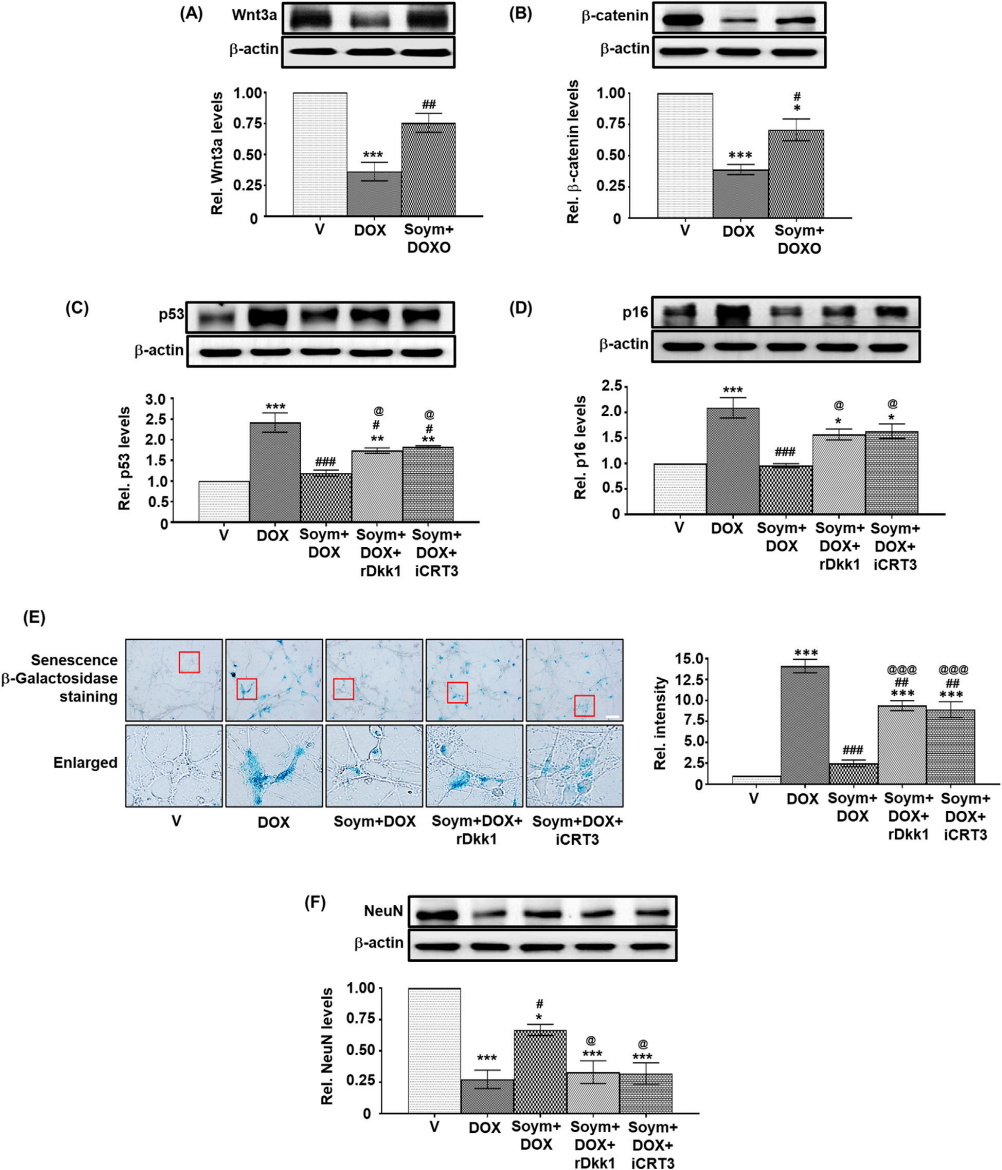
*Soymetide inhibits the doxorubicin-induced reduction in Wnt3a, β-catenin, and NeuN and the increase in senescence of primary mice hippocampal neurons*. Primary hippocampal neurons treated with vehicle (V), doxorubicin (DOX), Soymetide (Soym)+DOX, Soym + DOX + rDkk1 and Soym + DOX + iCRT3 were harvested. **(A–D, F)** Representative Western blots and corresponding densitometry (relative to vehicle) of Wnt3a **(A)**, β-catenin **(B)**, p53 **(C)**, p16 **(D)** and NeuN **(F)** normalized with β-actin in primary hippocampal neurons. **(E)** Representative photomicrographs and corresponding bar graph (relative to vehicle) of Senescence β-Galactosidase-stained primary hippocampal neurons. Scale bar: 20 µm. Data represent means ± SE of three independent experiments. ***p < 0.001, **p < 0.01 and *p < 0.05 compared to V. ^###^p < 0.001, ^##^p < 0.01 and ^#^p < 0.05 compared to DOX. ^@@@^p < 0.001 and ^@^p < 0.05 compared to Soym + DOX.

**FIGURE 7:**
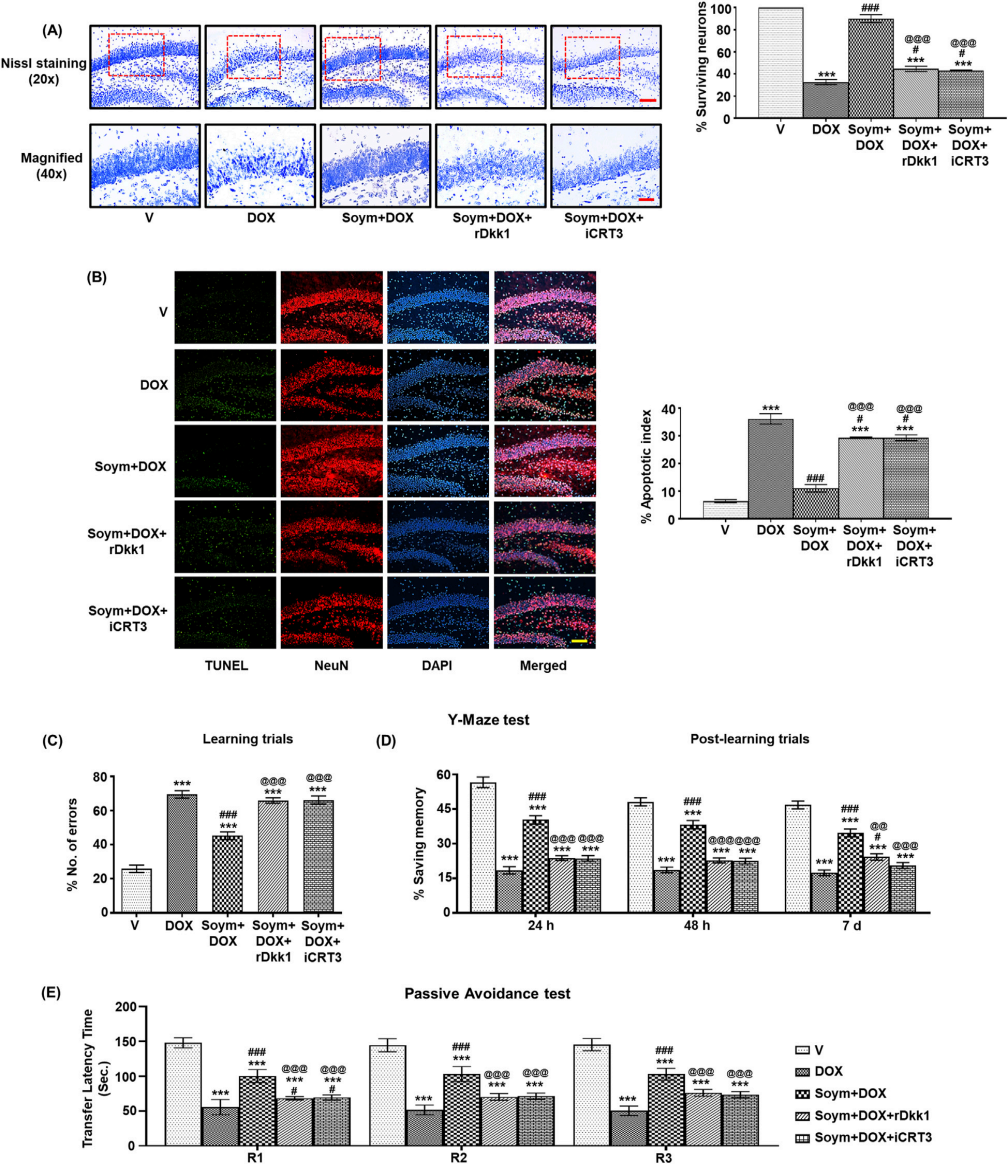
*Soymetide protects against doxorubicin-induced hippocampal neuronal loss and learning-memory impairment via the Wnt/β-catenin pathway in mice*. Brain sections were made from vehicle (V), doxorubicin (DOX), Soymetide (Soym)+DOX, Soym + DOX + rDkk1 and Soym + DOX + iCRT3-treated mice. **(A)** Upper panel-20x (with quantification, %) and lower panel-40x represent photomicrographs of Nissl-stained hippocampal neurons. Scale bar: 100 µm (20X) and 30 µm (40X). **(B)** Representative fluorescence photomicrographs of TUNEL, immunolabeled with NeuN and counter-stained with DAPI in the hippocampus. Bar diagrams represent corresponding quantification relative to the vehicle. Scale bar: 100 µm. Y -Maze and Passive Avoidance tests were performed in vehicle (V), doxorubicin (DOX), Soymetide (Soym)+ DOX, Soym + DOX + rDkk1 and Soym + DOX + iCRT3-treated mice. **(C, D)** Representative bar graphs for the number of errors (%) during learning trials **(C)** and the memory retained (% saving memory) at 24 h, 48 h and 7-day post-learning **(D)** in Y-Maze test. **(E)** Representative bar graphs for TLT for first (R1), second (R2), and third (R3) retention trials (24 h, 48 h, and 72 h, respectively) in Passive Avoidance test. Data represent means ± SE of three mice/group **(A, B)** and seven mice/group **(C–E)**. ***p < 0.001 compared to V. ^###^p < 0.001 and ^#^p < 0.05 compared to DOX. ^@@@^p < 0.001 and ^@@^p < 0.01 compared to Soym + DOX.

One-way ANOVA for comparisons involving a single parameter across multiple groups (Figures 1A–7C) or two-way ANOVA for multiple parameters and groups (Figures 7D, E), with subsequent Tukey’s posthoc tests for detailed comparisons, was conducted (GraphPad Software, Inc., San Diego, California).

## Results

### Effect of Soymetide and doxorubicin on senescence markers in the hippocampus of mice

We conducted a screening study to investigate how different doses of doxorubicin (1 mg/kg, 5 mg/kg, and 10 mg/kg) affect senescence in the hippocampus of mice. Specifically, we measured the levels of senescence markers p53, p21 and p16. Our observation indicated that 1 mg/kg had no effect, while 5 mg/kg elevated the hippocampal p53 (Figure 1A), p21 (Figure 1B), and p16 (Figure 1C) levels, and 10 mg/kg was lethal (data not shown). Therefore, we continued our study using a 5 mg/kg dose of doxorubicin. Next, we investigated whether Soymetide pretreatment (10, 50, and 100 μg/kg) affected the doxorubicin-induced senescence in the hippocampus of mice. Our findings indicated that 10 μg/kg of Soymetide had no effect. However, doses of 50 and 100 μg/kg caused a reduction in the doxorubicin-induced hippocampal p53 (Figure 1D), p21 (Figure 1E) and p16 (Figure 1F) levels, which were comparable to that of a previously demonstrated senolytic combination, D + Q (referred to Seno in Figure) (Krzystyniak et al., 2022) (Figures 1D–F). (The scrambled peptide did not affect the doxorubicin-induced senescence in the hippocampus (Supplementary Material 3). We conducted further analysis of the other features of senescence. We found that doxorubicin increased the expression of SASP inflammatory factors, such as IL-1α, IL-1β and IL-6, while Soymetide (50 and 100 μg/kg) and senolytic combination, D + Q reduced SASP levels (Figure 2A). We proceeded with our study using 50 μg/kg of Soymetide, i.e., the lower dose of Soymetide showing anti-senescence activity.

We further found that doxorubicin reduced the matured neuronal marker, NeuN (Figure 2B), and increased the Senescence β-Galactosidase staining in neurons (Figure 2C). These effects were inhibited by Soymetide (Figures 2B, C).

### Effect of Soymetide and doxorubicin on Wnt pathway and its link with anti-senescence effects in the hippocampus

We explored the effects of doxorubicin on the Wnt pathway (known to participate in neuronal functioning (Garg and Bandyopadhyay, 2024) and its involvement in the anti-senescence activity induced by Soymetide. Our initial findings demonstrated a reduction in the levels of hippocampal Wnt3a (Figure 3A), along with decreases in Wnt receptors, p-LRP6 (Figure 3B) and Fz (Figure 3C), and Wnt pathway components, Dvl (Figure 3D) and Axin1 (Figure 3E), following administration of doxorubicin. This treatment caused an increase in GSK-3β levels (Figure 3F) and a decrease in β-catenin (Figure 3G). However, pre-treatment with Soymetide appeared to counteract these effects, suggesting a restoration of the Wnt/β-catenin pathway (Figures 3A–G). Upon investigating the interplay between the Wnt/β-catenin pathway components and anti-senescence mechanism, our findings demonstrated that blocking Wnt and β-catenin with rDkk1 and iCRT3, respectively, prevented the Soymetide-induced decreases in p53 (Figure 4A), p21 (Figure 4B) and p16 (Figure 4C) and Senescence β-Galactosidase staining (Figure 4D), and increase in NeuN levels (Figure 4E). These findings suggested the participation of the components of Wnt3a/β-catenin pathway in the senescence mechanism of the hippocampus, which Soymetide can inhibit.

### Effect of Soymetide and doxorubicin on senescence and Wnt signaling in the hippocampal neurons

We verified the involvement of Wnt3a/β-catenin signaling in the hippocampal neuronal senescence. Our co-immunolabeling experiment with NeuN revealed a decrease in Wnt3a (Figure 5A) and β-catenin (Figure 5B), and an increase in p53 (Figure 5C) and p16 (Figure 5D) in the hippocampal neurons of mice following doxorubicin treatment. However, these changes were counteracted by Soymetide (Figures 5A–D). Moreover, rDkk1 and iCRT3 inhibited the Soymetide-induced decrease in p53 (Figure 5C) and p16 (Figure 5D), thereby verifying that Soymetide exerts its anti-senescence effects by modulating the Wnt3a/β-catenin pathway in the hippocampal neurons.

We conducted *in vitro* validation of the anti-senescent mechanism of Soymetide (10 nM, Supplementary Material 2) in primary cultures of mouse hippocampal neurons. Consistent with the *in vivo* observations, our findings showed that doxorubicin treatment led to a decrease in the Wnt3a (Figure 6A) and β-catenin levels (Figure 6B), while elevating p53 (Figure 6C), p16 (Figure 6D) and Senescence β-Galactosidase staining (Figure 6E), and decreasing NeuN levels (Figure 6F). However, the doxorubicin-induced changes in Wnt3a , β-catenin and senescent markers were mitigated by Soymetide (Figures 6A–F). (The scrambled peptide failed to affect the doxorubicin-induced senescence **(** Supplementary Material 4). Additionally, these Soymetide-induced effects were blocked by rDkk1 and iCRT3 (Figures 6C–F). Hence, our findings verified the involvement of components of Wnt3a/β-catenin pathway in the hippocampal neuronal senescence and demonstrated the anti-senescence effect of Soymetide.

### Effect of Soymetide and doxorubicin on the hippocampal neuronal survival and learning-memory performance of mice

Wnt pathway and senescence are linked with neuronal survival and subsequent cognitive functions (Garg and Bandyopadhyay, 2024). Hence, we assessed the effect of doxorubicin and Soymetide on hippocampal Nissl staining for neuronal count, TUNEL staining for neuronal apoptosis, and learning-memory performance of mice. We found that doxorubicin led to a reduction in Nissl staining (Figure 7A) and increase in TUNEL positive neuronal apoptotic index (Figure 7B) in the hippocampus, indicating neuronal loss. However, Soymetide counteracted these effects (Figures 7A, B). Additionally, we observed that doxorubicin impaired learning and memory abilities, as demonstrated by an increased number of errors during learning trials (Figure 7C) and diminished memory retention in the Y-Maze test at various intervals post-learning (Figure 7D). Conversely, Soymetide treatment facilitated a notable improvement in these cognitive functions (Figures 7C, D). This was further supported by results from the Passive Avoidance test, where Soymetide enhanced the TLT across the retention trials in doxorubicin-treated mice (Figure 7E). The beneficial outcomes associated with Soymetide administration were counteracted by rDkk1 and iCRT3 (Figures 7A–E), suggesting that the neuroprotective and cognitive-enhancing effects of Soymetide are mediated through the components of the Wnt3a/β-catenin pathway.

## Discussion

**FIGURE 8:**
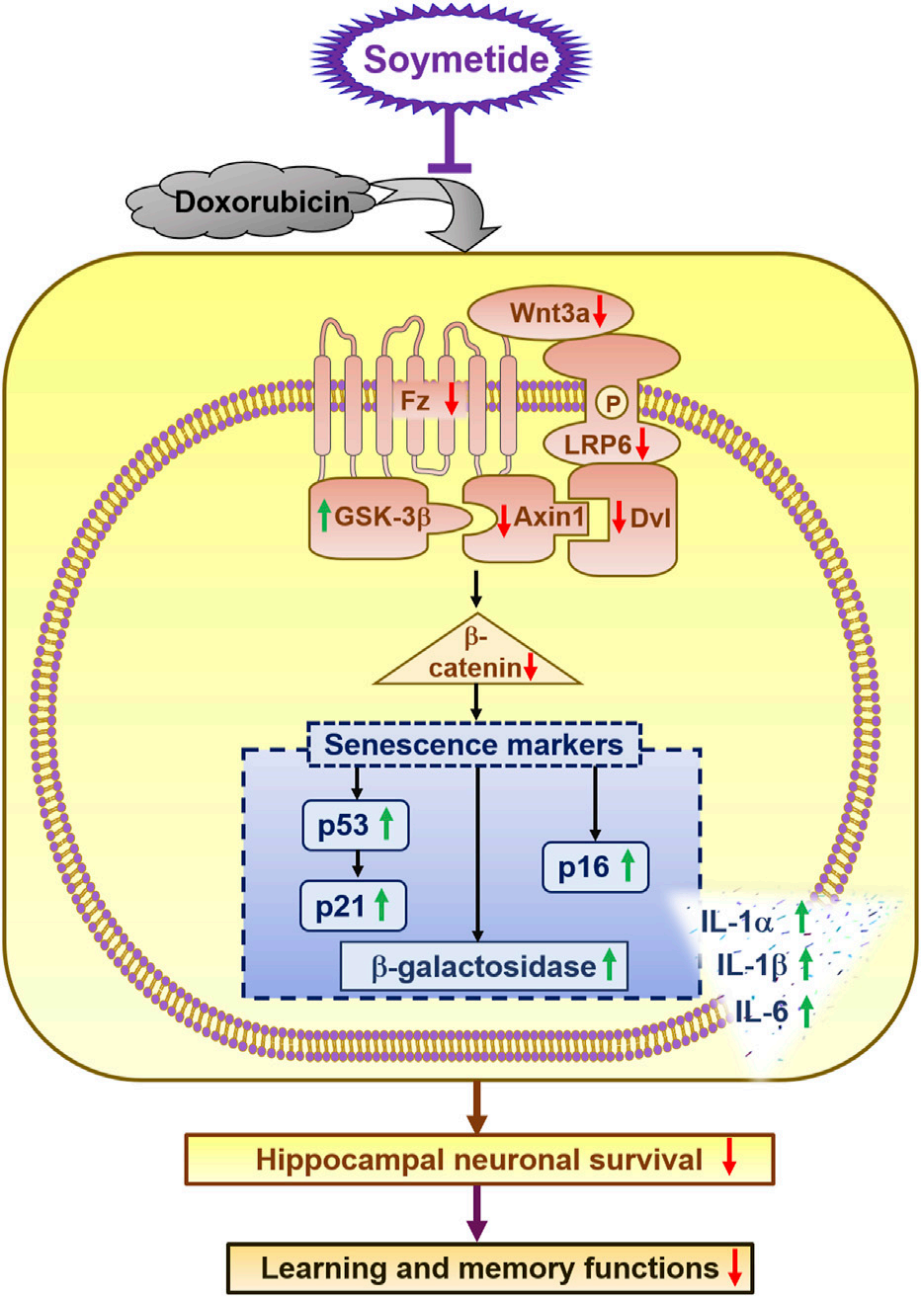
*Schematic of the anti-senescent effect induced by Soymetide against doxorubicin treatment in hippocampal neurons and its positive impact on cognitive function*. Soymetide prevents the reduction in Wnt3a and its related proteins (Fz, p-LRP-6, Dvl, and Axin1) caused by doxorubicin, and it counters the rise in GSK-3β and the reduction of β-catenin. This inhibits the increase in senescence markers such as p53, p21, p16, β- Galactosidase activity, and SASPs, as well as the decrease in neuronal survival and learning-memory functions. In summary, Soymetide exhibits anti-senescent effects, provides neuroprotection, and induces cognitive restoration by restoring the Wnt/β-catenin pathway.

The current study unravels novel neuroprotective properties of Soymetide, with a specific focus on: 1. Reduction of senescence in the hippocampus, with effects comparable to a known senolytic combination (D + Q). 2. Beneficial impact involving the Wnt/β-catenin pathway. The reduction of key components in the Wnt3a/β-catenin pathway accelerates neuronal senescence. However, Soymetide’s ability to restore Wnt/β-catenin signaling helped counteract this senescence process. 3. Improvement in neuronal survival and enhancement of learning and memory functions. The study overall introduces a soy peptide that shows promise for improving hippocampal health and cognitive functions by targeting the interlinked senescence pathways and Wnt/β-catenin mechanism, offering a potential therapeutic approach against cognitive decline (Figure 8).

Studies suggest that fermented soy food products like cheonggukjang and doenjang offer neuroprotection by inhibiting neuronal death, inflammation, and susceptibility to ischemic injury (Ko et al., 2019; Jeong et al., 2020; Jang et al., 2021). Nattō, a fermented whole soybean improves age-related cognitive decline and associated deregulated hippocampal neuronal signaling in senescence-accelerated mouse-prone eight mice (Zheng et al., 2023). Additionally, soy isoflavones (genistein and daidzein) and their metabolites (equol), and soyasaponin I (a triterpenoid compound) prevent neuronal apoptosis, promote neuroregeneration, synaptic plasticity, neurotransmission, and antioxidative effects (Ma et al., 2010; Hong et al., 2013; Lu et al., 2021; Shi et al., 2023). However, the soy-derived compounds may face limitations regarding their “drug-likeness” due to concerns about metabolic stability restricting potential therapeutic applications, as reported for phytochemicals (Sharan et al., 2009). Reports further indicate that soy peptides, VHVV and Tyr-Pro, enzymatic and fungal hydrolysates of soy proteins, respectively, maintain neuronal survival and reduce memory loss (Ju et al., 2019; Tanaka et al., 2020). However, while hydrolyzed proteins and peptides show promise as food and potential nutraceutical applications, standardizing them as therapeutic products may pose challenges. In this context, Soymetide, a well-characterized small/short soy-derived peptide can serve as a promising biologic/peptide drug, marking a considerable advancement in this field. Our findings identify Soymetide as a neuroprotectant that promotes a wide range of anti-senescent features in the hippocampal neurons, comparable with broad-target senolytic drugs (D + Q). The small peptide is proposed to hold potential for advancing peptide drug design and development, based on probable advantages such as improved absorption, enhanced cell targeting, reduced immunogenicity, and increased cost-effectiveness (Fosgerau and Hoffmann, 2015). These features may position Soymetide as a potential candidate for therapies aimed at anti-senescence and neuroprotection. Besides, this novel pro-cognitive role adds a notable new dimension to the functional profile of Soymetide, reported for immunostimulating and anti-cancer activities (Schuler et al., 1982; Tsuruki et al., 2003), offering fresh insights into its potential mechanism of action. The selected dose of 50 μg/kg of Soymetide was identified for its effective anti-senescence properties, offering a cost-effective alternative to the higher 100 μg/kg dose. While safety data was not specifically assessed in this study, the lower dose presents a promising approach that warrants further investigation to determine the best balance of safety and therapeutic benefits.

Prior studies show that eliminating p16-expressing senescent cells and lowering the p53 stress protein can prevent mild cognitive impairment, neuronal death, and synaptic issues (Bussian et al., 2018; Zhang et al., 2022b). This process minimizes p53-triggered p21 activation and DNA damage, averting cell cycle arrest and senescence (de Carne Trecesson et al., 2011; Tajima et al., 2019; Tufekci et al., 2021). Our research suggests that Soymetide influences these communications involving p16, p21, p53, and SASPs, leading to healthier neuronal expression, reduced cellular damage, and improved brain repair, benefiting the central nervous system (CNS). Specifically, regarding soy peptides, previous research has mainly focused on their roles in preventing and suppressing tumors by engaging pathways such as p53, p16, p21, and cytokines, and growth arrest and apoptosis in various cancer cells (Lin et al., 1998; Payton-Stewart et al., 2009). Studies in osteoblasts did show the involvement of p53/p21 pathway in bone development following soy peptide treatment (Zhang et al., 2014; Chen et al., 2015). However, the lack of research on the impact of soy peptides on the brain, specifically its anti-senescent effects on the hippocampus, underscores the importance and promise of our study on these soy-based peptides for future exploration.

Our research enriches the current understanding of senescence in the hippocampal neurons and extends upon earlier work (Bayod et al., 2015; Su et al., 2018), which showed a link between the upregulation of the Wnt pathway inhibitor, Dickkopf-1, and the perturbation of essential elements within the Wnt/β-catenin signaling cascade. Our findings further detail that the senescence process is associated with precise alterations in Wnt signaling markers in the hippocampal neurons, which correspond with an apparent decline in neuronal viability, as evidenced by a diminished reduction in Nissl bodies, along with NeuN expression. In a notable progression from past research (Fortress and Frick, 2016; Arredondo et al., 2020), which predominantly focused on the broader aspects of Wnt signaling, our study also identifies a decrease in both the Wnt ligands and its corresponding Frizzled receptor during senescence. Moreover, while prior reports only noted changes in Wnt signaling during hippocampal senescence (Bayod et al., 2015; Fortress and Frick, 2016), our investigation additionally sheds light on how these modifications in Wnt pathway actively played a key role in controlling neuronal senescence and the impact on its essential molecules, such as p53, p21, p16, SASP, β-Galactosidase, etc. Our research explicitly linked these observations with Soymetide treatment, demonstrating its restoration in the levels of the Wnt ligand and Frizzled receptor in the senescent neurons. Moreover, it unraveled that Soymetide’s anti-senescent effects are highly dependent on regulated Wnt signaling, demonstrated by the use of the Wnt antagonist rDkk1 and the β-catenin inhibitor iCRT3, which blocked the reduction of senescent markers in the hippocampal neurons. In this context, in terms of soy-based protein and Wnt signaling (as such), there have been several contradictory reports. On the one hand, soy proteins (predominantly genistein) reduced β-catenin signaling (via the participation of altered IGF-1 and E-cadherin production) leading to a decreased T-cell factor/lymphoid enhancer factor (TCF/LEF) activity and resulting prostate cancer cell growth, development of hepatocellular carcinoma, intestinal tumorigenesis, and proliferation in cancer cells (Liss et al., 2010; Mahmoud et al., 2014; Mercer et al., 2017). Other studies indicated that genistein and soy isoflavones enhanced Wnt/β-catenin signaling and led to osteoblast formation, improved bone health, and prevented non-alcoholic fatty liver disease (NAFLD) and pathological adiposity (Kim and Kang, 2012; Mannino et al., 2024). In line with the latter, results from our study demonstrated the neuroprotective effects of Soymetide through the enhancement of the components of the Wnt/β-catenin pathway, verifying a protective role of soy peptides in increasing Wnt/β-catenin pathway for normal cells as opposed to the tumorous and cancerous cells. As a supportive observation, Soymetide inhibits the reduction of Transcription Factor 3 (TCF3) levels caused by doxorubicin (Supplementary Material 5), which emphasize the involvement of the Wnt/β-catenin pathway. This effect could be linked to the regulation of p53, p21, p16, and β-galactosidase, laying the groundwork for future studies to investigate its mechanisms of action. Hence, it may be said that in terms of the Wnt pathway, the importance of our study for hippocampal neurons is two-fold. It offers new insights into the senescence pathway and emphasizes the potential of Soymetide in mitigating the adverse effects of senescence.

Research indicates that soy proteins, isoflavone extracts, and peptides can enhance cognitive performance in adults, postmenopausal women, and the aged by activating brain pathways such as Brain-Derived Neurotrophic Factor (BDNF)-Tropomyosin-related kinase receptor B (TrkB)/cAMP response element-binding protein (CREB) signaling, N-methyl-D-aspartate receptor (NMDAR)-calcium/calmodulin-dependent protein kinase II (CaMKII) cascade (NMDAR-CaMKII), as well as Phosphatidylinositol-3 Kinase (PI3K)/Akt signaling, and through antioxidant and cholinergic properties (Kreijkamp-Kaspers et al., 2004; Geller and Studee, 2006; Katayama et al., 2014; Unno and Konishi, 2015; Yoo et al., 2022; Zheng et al., 2023). Earlier individual studies have also shown a connection between Wnt/β-catenin signaling with the BDNF (TrkB)/CREB signaling, NMDAR-CaMKII, PI3K/Akt, antioxidant, and cholinergic properties (Arrazola et al., 2009; Sinha et al., 2015; Xu et al., 2015; Hu et al., 2019; Ishidori et al., 2022; Nachtigall et al., 2023; Lou et al., 2024). Hence, in continuation, our research highlights the importance of investigating the relationships between neurotrophins, neurotransmitters, antioxidants, and specific signaling pathways in Sometide-treated hippocampal neurons. Our work points to the need to explore Soymetide’s impact on aging, specifically concerning hippocampal Wnt signaling. Moreover, while our study demonstrated Soymetide’s anti-senescence effects when directly applied to the hippocampus, proving its potential to protect against neuronal aging, this method is infeasible for human use. Therefore, there is a need for further research to develop a brain-transportable Soymetide formulation for systemic administration.

Hence, our research suggests that Soymetide has the potential to diminish cellular senescence in hippocampal neurons, which may improve cognitive performance and maintain neuronal health through modulation of senescence markers. Additionally, we propose that Soymetide might possess anti-inflammatory properties through its impact on macrophages and monocytes, offering neuroprotection against age-related diseases. The observed reduction in serum pro-inflammatory cytokines suggests an immune modulation mechanism that warrants further exploration.

We recognize few limitations in this study. The primary focus has been on investigating the effectiveness of Soymetide as a single-agent treatment. Additionally, while the potential for immune modulation has not been thoroughly examined, and the use of combination therapies (pairing Soymetide with dasatinib or quercetin) has yet to be explored, these aspects are crucial for future research aimed at improving treatment efficacy and minimizing toxicity. Furthermore, it is vital to determine optimal dosages, study the pharmacokinetics of Soymetide in various biological settings, and develop more effective peptide variants through *in vitro* assays, with the goal of extending these findings to *in vivo* applications. These efforts will enhance our understanding and optimize the therapeutic outcomes when utilizing Soymetide.

## Data Availability

The raw data supporting the conclusions of this article will be made available by the authors, without undue reservation.
