# Supplementary figures and images for: Exploring the potential anti-senescence effects of soybean-derived peptide Soymetide in mice hippocampal neurons via the Wnt/β-catenin pathway
Source: Front Pharmacol. 2025 Feb 25;16:1510337. doi: 10.3389/fphar.2025.1510337 (PMC11893861; doi:10.3389/fphar.2025.1510337)

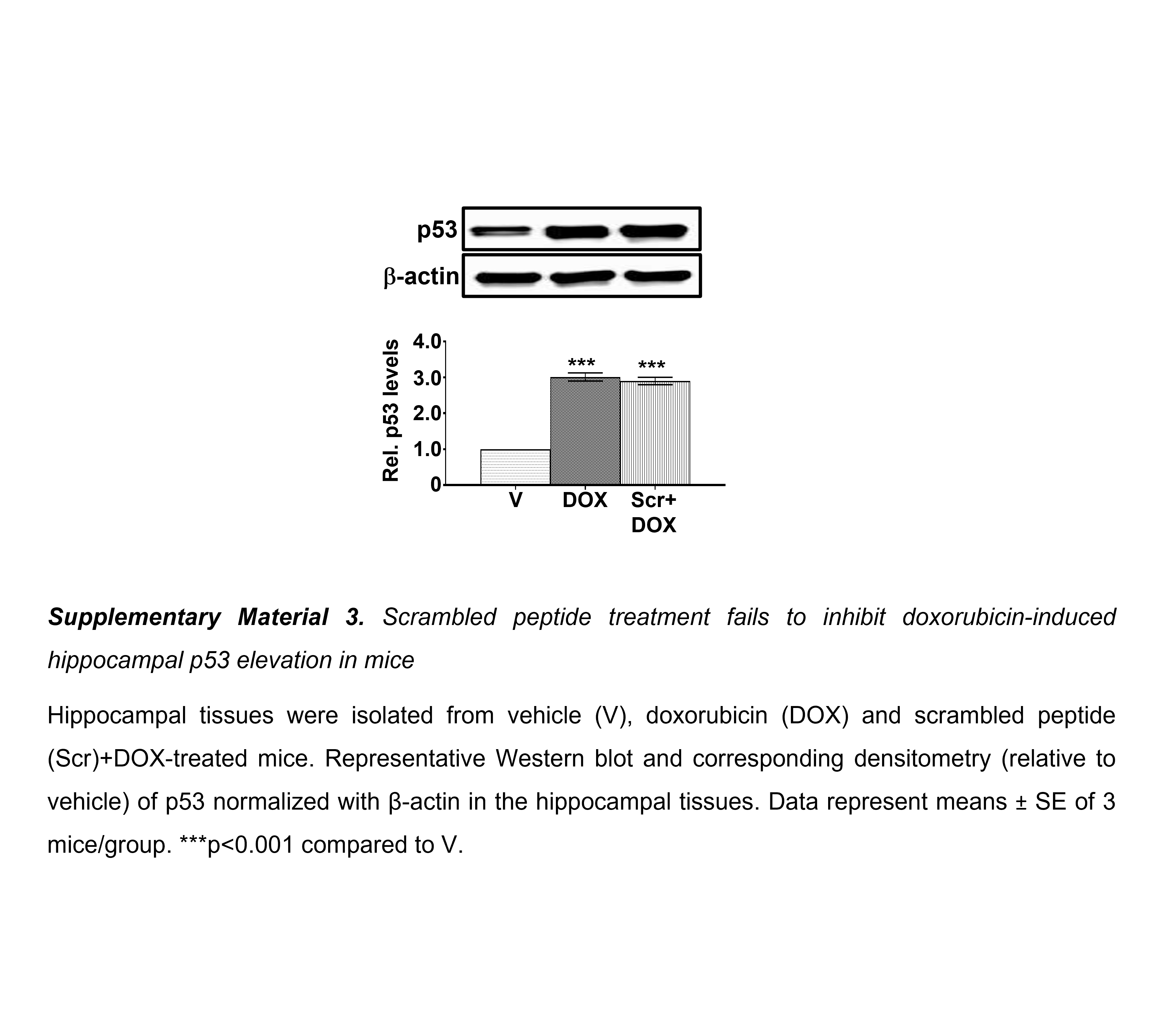

Supplement: Supplementary file 1 [file Image3.tif]

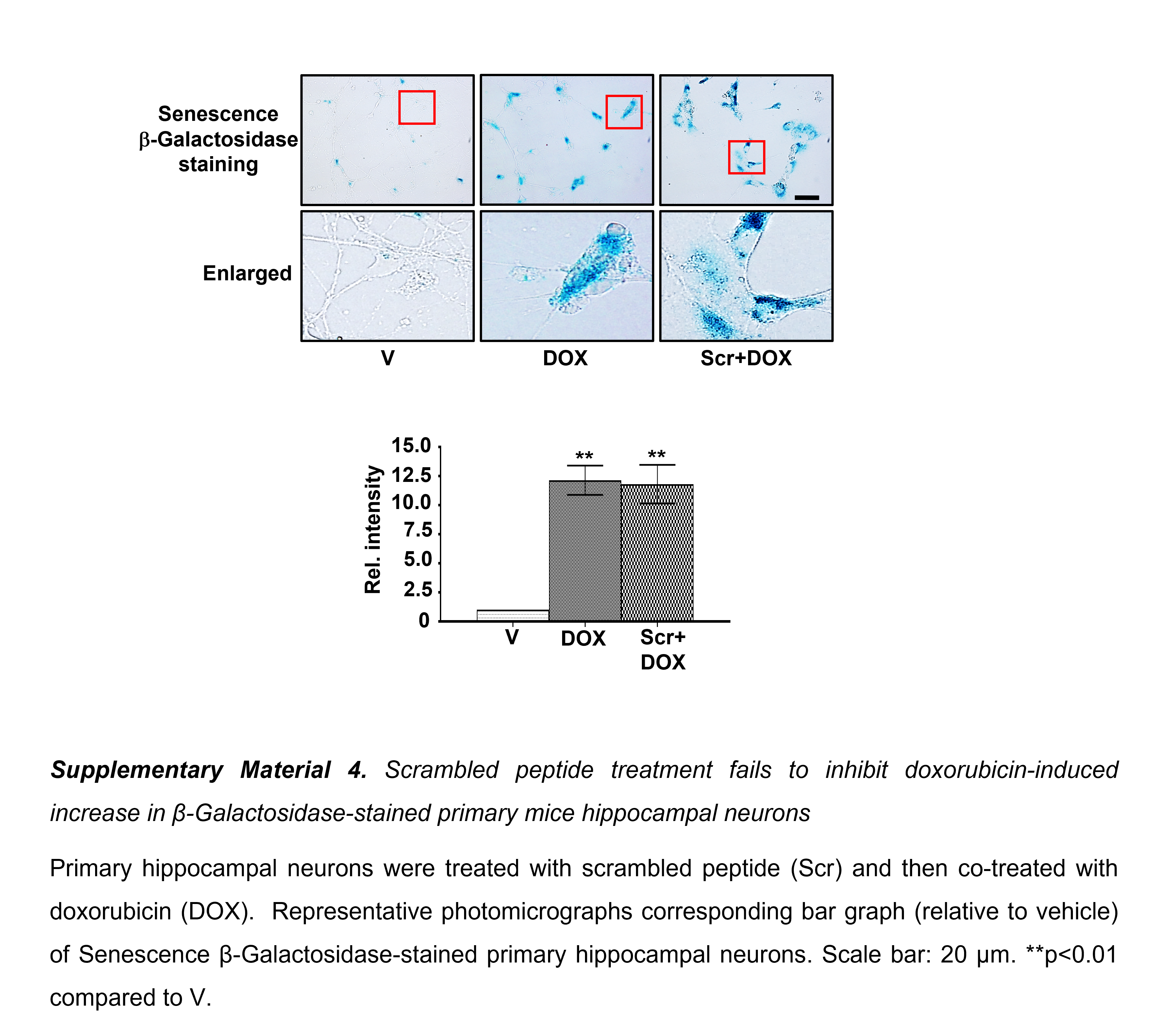

Supplement: Supplementary file 2 [file Image4.tif]

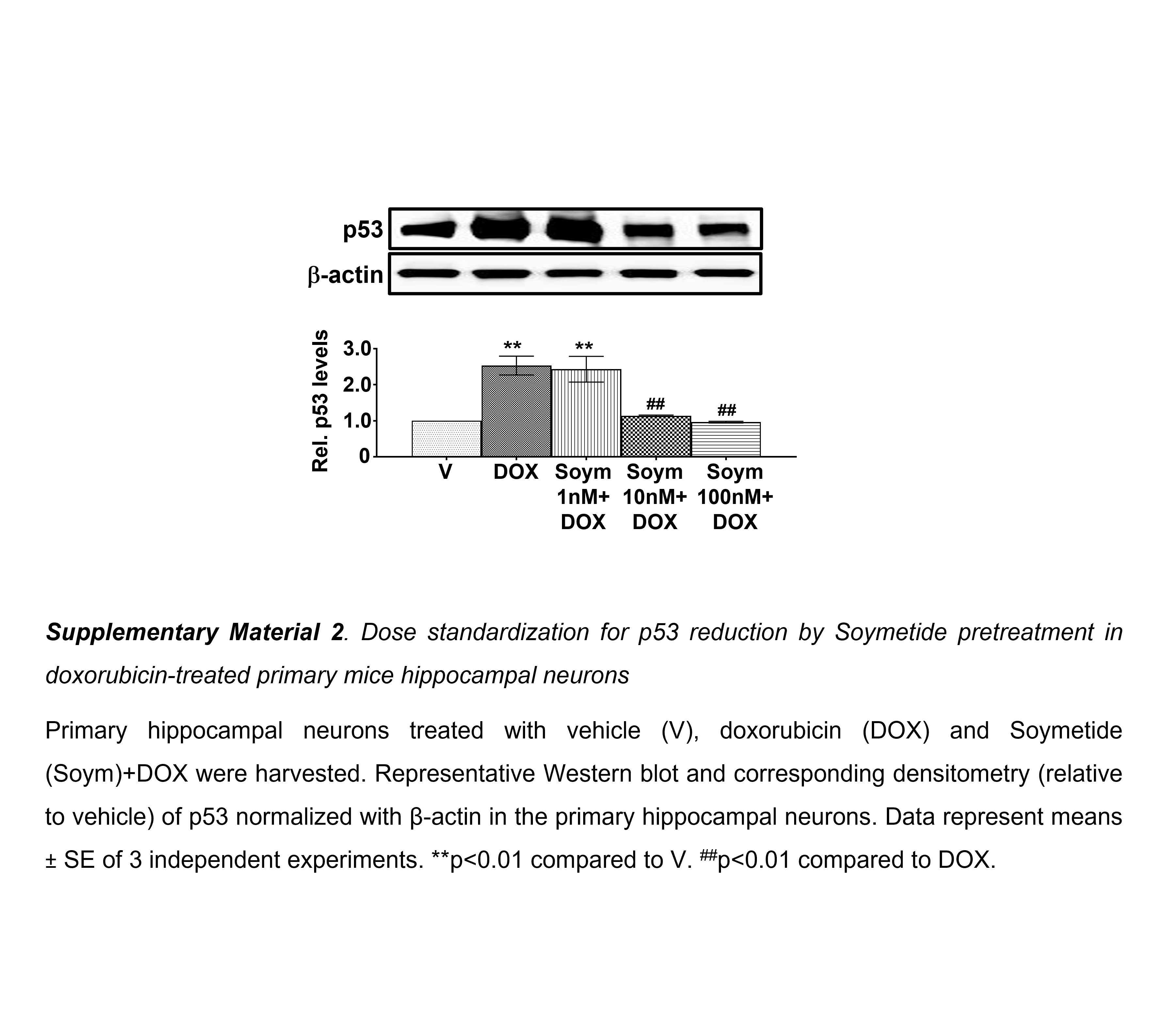

Supplement: Supplementary file 3 [file Image2.tif]

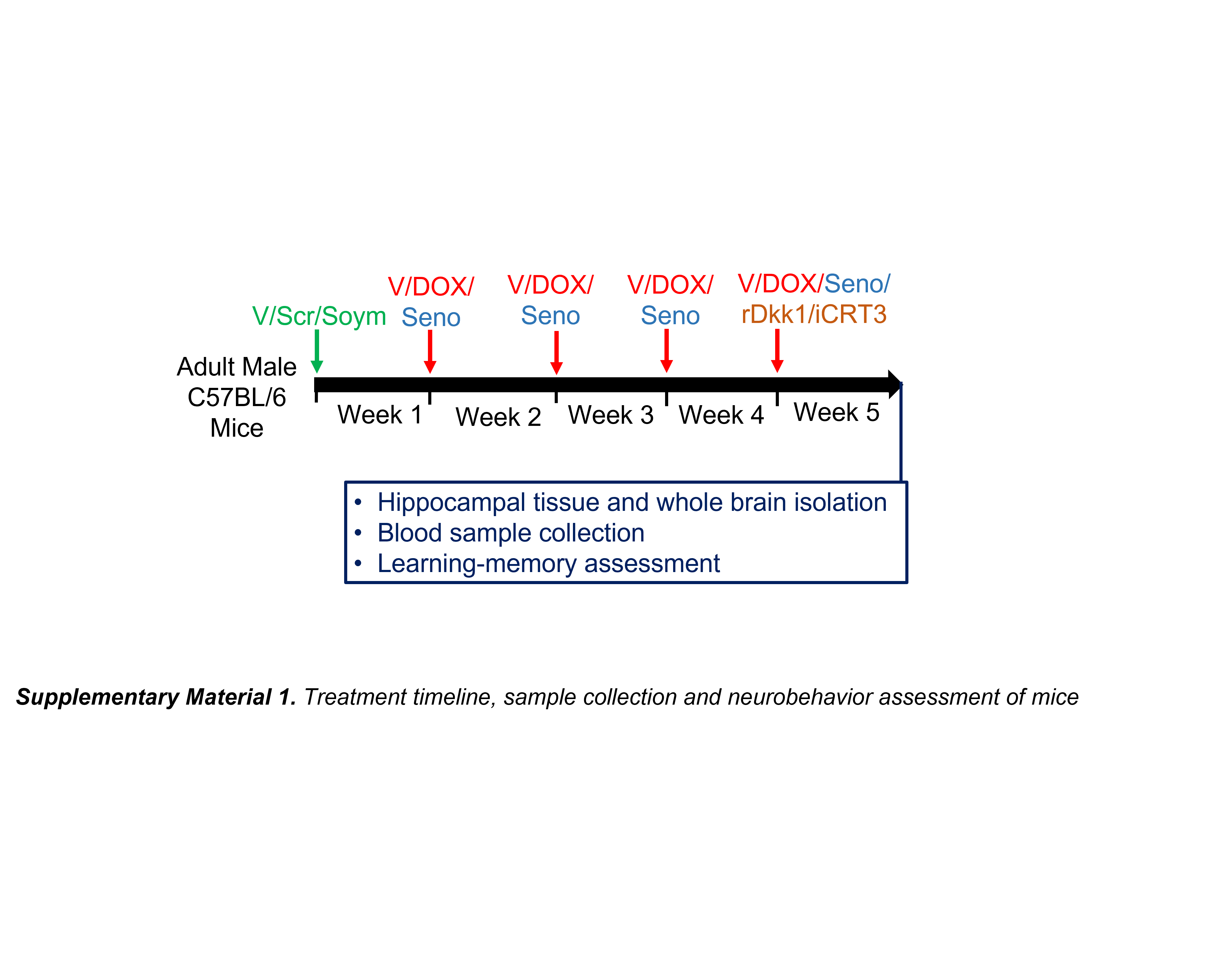

Supplement: Supplementary file 4 [file Image1.tif]

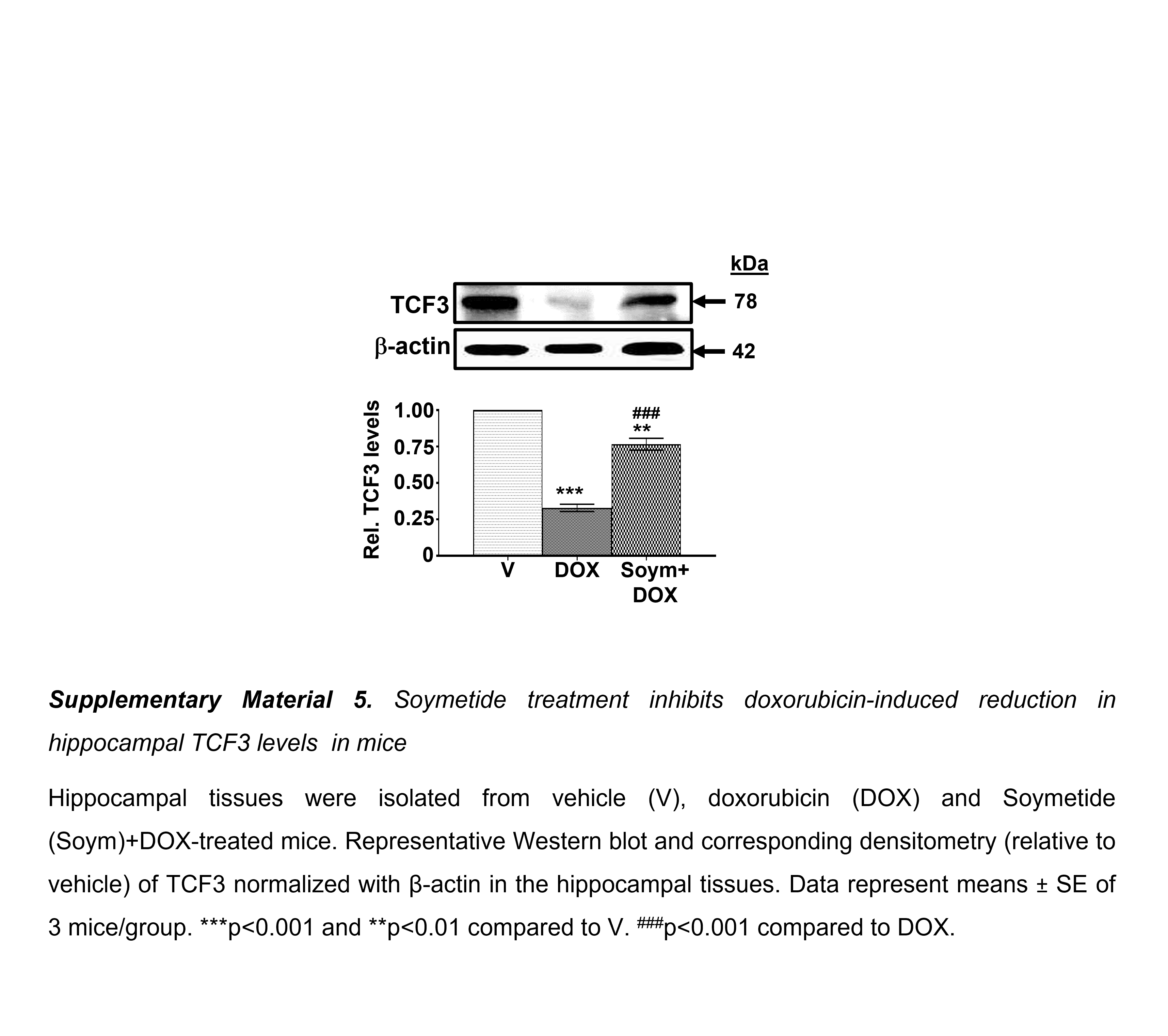

Supplement: Supplementary file 5 [file Image5.tif]
